# Supplementary material for: Mechanisms governing the pioneering and redistribution capabilities of the non-classical pioneer PU.1
Source: Nat Commun. 2020 Jan 21;11:402. doi: 10.1038/s41467-019-13960-2 (PMC6972792; doi:10.1038/s41467-019-13960-2)
Supplement: Supplementary file 7 — Source data [file 41467_2019_13960_MOESM7_ESM.zip › Source_Data/Figure6/Figure6B_MotifScanOutput/ETS-specific/homerResults/motif1.similar.html]

motif1

## Information for motif1

C
T
G
A
T
G
A
C
G
T
A
C
A
C
T
G
A
C
T
G
C
G
T
A
C
G
T
A
C
T
A
G
A
G
C
T
T
C
A
G
T
C
A
G
A
T
G
C
  
Reverse Opposite:  

T
A
C
G
A
G
T
C
A
G
T
C
T
C
G
A
A
G
T
C
C
G
A
T
A
C
G
T
A
G
T
C
A
G
T
C
A
C
T
G
A
C
T
G
G
A
C
T
  

|  |  |
| --- | --- |
| p-value: | 1e-1757 |
| log p-value: | -4.047e+03 |
| Information Content per bp: | 1.600 |
| Number of Target Sequences with motif | 5574.0 |
| Percentage of Target Sequences with motif | 72.82% |
| Number of Background Sequences with motif | 7946.1 |
| Percentage of Background Sequences with motif | 19.69% |
| Average Position of motif in Targets | 102.0 +/- 42.2bp |
| Average Position of motif in Background | 100.5 +/- 61.8bp |
| Strand Bias (log2 ratio + to - strand density) | -0.0 |
| Multiplicity (# of sites on avg that occur together) | 1.38 |
| Motif File: | file (matrix) reverse opposite |

### Similar de novo motifs found

|  |  |  |  |  |  |  |  |
| --- | --- | --- | --- | --- | --- | --- | --- |
| Rank | Match Score | Redundant Motif | P-value | log P-value | % of Targets | % of Background | Motif file |
| 1 | 0.971 | A G T C T C A G A G T C C G A T C G A T T A G C G A T C A C T G A T C G G A C T | 1e-1740 | -4008.790680 | 70.93% | 18.46% | motif file (matrix) |
| 2 | 0.969 | T C G A T A G C T G A C A C T G T C A G C G T A G C T A T C A G A G C T | 1e-1620 | -3732.280198 | 66.86% | 16.92% | motif file (matrix) |
| 3 | 0.994 | T C G A T A G C T G C A A C T G T C A G T G C A C G T A T C A G A G C T T C A G T C A G T A G C C T A G | 1e-1588 | -3657.516323 | 67.62% | 17.85% | motif file (matrix) |
| 4 | 0.932 | G T A C A C T G A C T G C G T A C G T A C T A G A G C T C A G T | 1e-1362 | -3137.268413 | 76.76% | 28.67% | motif file (matrix) |
| 5 | 0.906 | A G T C C G A T A C G T A G T C A G T C A C T G A T C G G A C T | 1e-1351 | -3111.439711 | 61.88% | 16.73% | motif file (matrix) |
| 6 | 0.925 | G T A C A C T G A C T G C G T A C G T A C T G A A G C T | 1e-1346 | -3099.372883 | 77.31% | 29.47% | motif file (matrix) |
| 7 | 0.934 | T C G A T A G C G T C A A C T G A C T G C G T A C G T A C T A G G A C T C T A G T C A G A T C G A C T G T C A G | 1e-1269 | -2923.054872 | 59.93% | 16.44% | motif file (matrix) |
| 8 | 0.891 | C G A T A C G T A G T C A G T C A C T G A C T G G A C T | 1e-910 | -2097.045139 | 52.53% | 16.38% | motif file (matrix) |
| 9 | 0.849 | T C A G A G C T A G C T C G A T G T A C T A G C A C T G A G C T A G C T | 1e-497 | -1145.939381 | 38.41% | 13.75% | motif file (matrix) |
| 10 | 0.833 | C T G A A G T C T G A C T C A G A T C G C G T A C G T A C T A G C T A G | 1e-354 | -817.300051 | 23.47% | 6.93% | motif file (matrix) |
| 11 | 0.754 | C T G A T A G C A T G C A T C G A T C G C G T A G C T A T C A G | 1e-326 | -751.450565 | 52.95% | 29.63% | motif file (matrix) |
| 12 | 0.835 | A T G C T A G C C A G T A C T G T C A G A G T C A C G T A C G T A G T C A G T C A C G T A C T G A G T C A G T C | 1e-208 | -479.262925 | 12.48% | 3.20% | motif file (matrix) |
| 13 | 0.826 | A C T G T C G A A G C T G C A T A C G T A G T C A G T C A C T G A G T C A G C T A T G C | 1e-106 | -244.383030 | 9.20% | 3.14% | motif file (matrix) |
| 14 | 0.758 | A T C G A G T C A C G T A G T C G T A C A C T G A C T G C G T A C G T A A C T G A C T G T G A C T A C G A T G C | 1e-64 | -148.239399 | 3.33% | 0.71% | motif file (matrix) |
| 15 | 0.794 | C G A T A T G C G T C A A G T C T G C A C T A G T A C G C G T A G T C A C T A G T G A C G T A C A C G T | 1e-56 | -130.993591 | 4.56% | 1.46% | motif file (matrix) |
